# Influence of optic disc-fovea distance on macular thickness measurements with OCT in healthy myopic eyes

**DOI:** 10.1038/s41598-018-23479-z

**Published:** 2018-03-27

**Authors:** Kunliang Qiu, Geng Wang, Riping Zhang, Xuehui Lu, Mingzhi Zhang, Nomdo M. Jansonius

**Affiliations:** 1 Joint Shantou International Eye Center of Shantou University and The Chinese University of Hong Kong, Shantou, Guangdong Province The People’s Republic of China; 2 Department of Ophthalmology, University of Groningen, University Medical Center Groningen, Groningen, The Netherlands; 3 Department of Epidemiology, Erasmus Medical Center, Rotterdam, The Netherlands

## Abstract

Assessment of macular thickness is important in the evaluation of various eye diseases. This study aimed to determine the influence of the optic disc-fovea distance (DFD) on macular thickness in myopic eyes. We determined the DFD and the macular thickness in 138 eyes from 138 healthy myopic subjects using the Cirrus HD-OCT. Correlation analysis and multiple linear regression were performed to determine the influence of DFD, axial length, disc area, and β-PPA on macular thickness. To further remove the confounding effect of ocular magnification on the DFD and OCT scan area, a subgroup analysis was performed in eyes with a limited axial length range (24–25 mm). DFD was significantly correlated with both regional (central, inner, and outer ETDRS subfields) and overall average macular thickness at a Bonferroni corrected P value of 0.004 (r ranging from −0.27 to −0.47), except for the temporal outer (r = −0.15, P = 0.089) and inferior outer (r = −0.22, P = 0.011) macular thickness. In the multivariable analysis, DFD was significantly associated with the average inner and outer macular thickness, the central subfield thickness, and the overall macular thickness (all P < 0.001), independent of ocular magnification and other covariates. Our findings indicate that eyes with a greater DFD have a lower macular thickness.

## Introduction

Myopia is a prevalent condition in Asia and a major risk factor for glaucoma and various forms of progressive maculopathy^1–8^. The introduction of optical coherence tomography (OCT) has facilitated the detection of glaucoma and myopic maculopathy^9–12^. While macular thickness measurements with OCT are useful to detect glaucoma and myopia associated maculopathy, significant variation of macular thickness in healthy individuals has confounded the detection of macular pathologies^13–25^.

In a population-based study, Gupta *et al*. reported that a longer axial length was significantly associated with a lower overall macular thickness as measured with spectral-domain OCT^17^. The distance between the optic disc center and the fovea (DFD) is another biometric variable that may influence the macular thickness. Myopic eyes are likely to have a large DFD; the elongation of the eyeball may stretch the fovea further away from the optic disc center^26^. It has been shown that the DFD is associated with the peripapillary retinal nerve fiber distribution in healthy eyes^27^. However, to our knowledge, the relationship between DFD and macular thickness has not been reported.

The objective of this study was to determine the influence of DFD on macular thickness. For this purpose, we performed spectral-domain OCT measurements in healthy myopic eyes.

## Results

**Figure 1:**
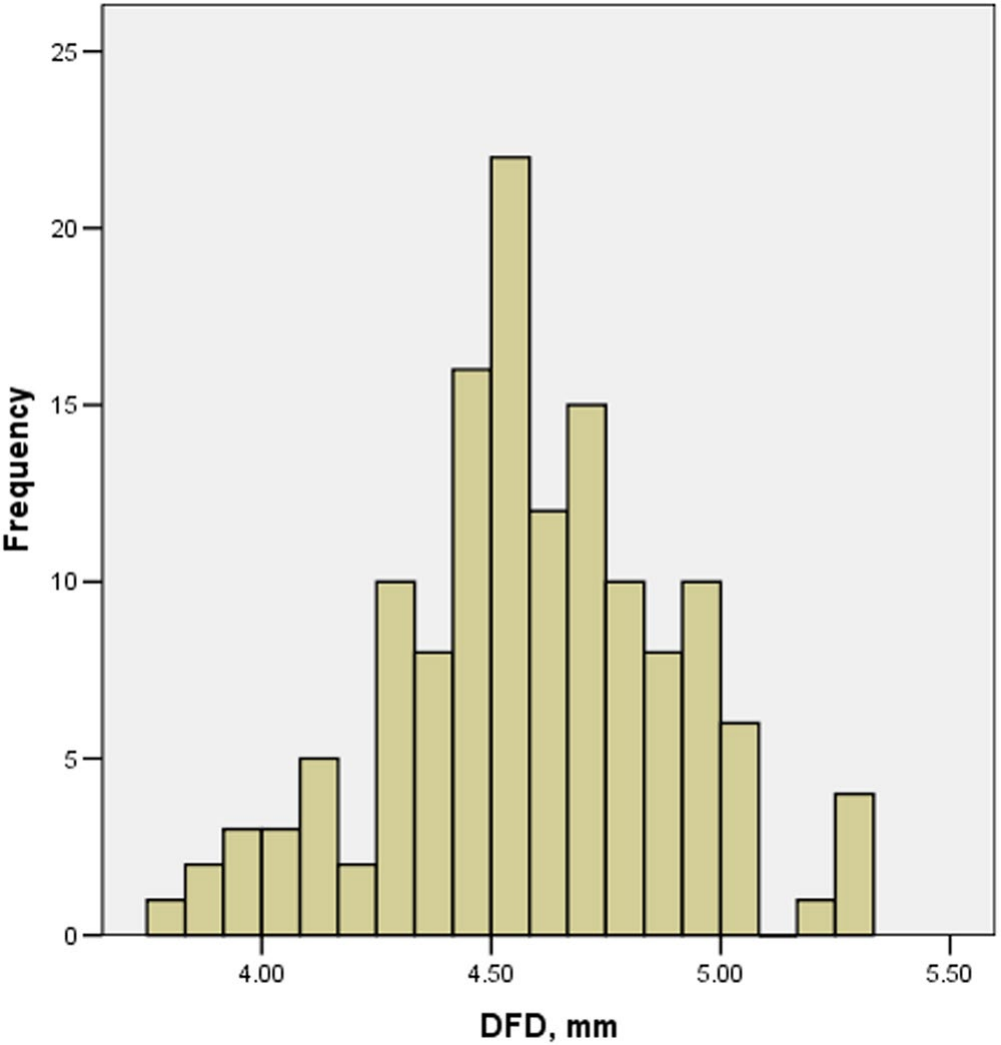
Histogram of disc-fovea distance (DFD) of all included eyes.

Nine subjects were excluded because of unreliable visual field tests (6 subjects) and poor OCT scan quality (3 subjects). As a result, we included 138 eyes from 138 subjects (60 females and 89 right eyes). Table 1 shows the demographics of the study population. The mean ± standard deviation refractive error and axial length were −5.12 ± 2.30 D and 25.57 ± 1.09 mm, respectively. The mean DFD was 4.58 ± 0.30 mm. Figure 1 displays the distribution of DFD across all subjects. The overall macular thickness and volume were 277.0 ± 12.1 μm and 9.98 ± 0.44 mm^3^, respectively. DFD was significantly associated with axial length (r = 0.45, P < 0.001) and disc area (r = −0.26, P = 0.002). Of all the included eyes, 49% had β-PPA. Of all scans, 90% had a signal strength of 8 or better (median value 9).

**Table 1:** Characteristics of the study population.

	Mean ± SD	Range
Age, y	23.0 ± 4.0	18 to 40
Refractive error, D	−5.12 ± 2.30	−0.50 to −9.63
Axial length, mm	25.57 ± 1.09	22.5 to 28.8
Visual field mean deviation, dB	−2.18 ± 1.03	−4.96 to 1.47
Signal strength	8.5 ± 0.8	7 to 10
DFD, mm	4.58 ± 0.30	3.8 to 5.3
Disc area, mm^2^	1.90 ± 0.51	0.92 to 3.63
Superior Inner thickness, μm	319.4 ± 14.8	273 to 359
Nasal inner thickness, μm	318.5 ± 17.2	241 to 363
Inferior inner thickness, μm	311.0 ± 14.2	271 to 346
Temporal inner thickness, μm	302.6 ± 13.5	267 to 339
Average inner macular thickness, μm	312.9 ± 14.2	271 to 348
Superior outer thickness, μm	274.9 ± 12.7	243 to 308
Nasal outer thickness, μm	297.9 ± 16.0	262 to 346
Inferior outer thickness, μm	264.9 ± 13.6	228 to 311
Temporal outer thickness, μm	256.6 ± 12.1	227 to 297
Average outer macular thickness, μm	273.6 ± 12.4	241 to 315
Central subfield thickness, μm	249.2 ± 16.1	197 to 306
Overall macular thickness, μm	277.0 ± 12.1	249 to 317

SD = standard deviation.

**Figure 2:**
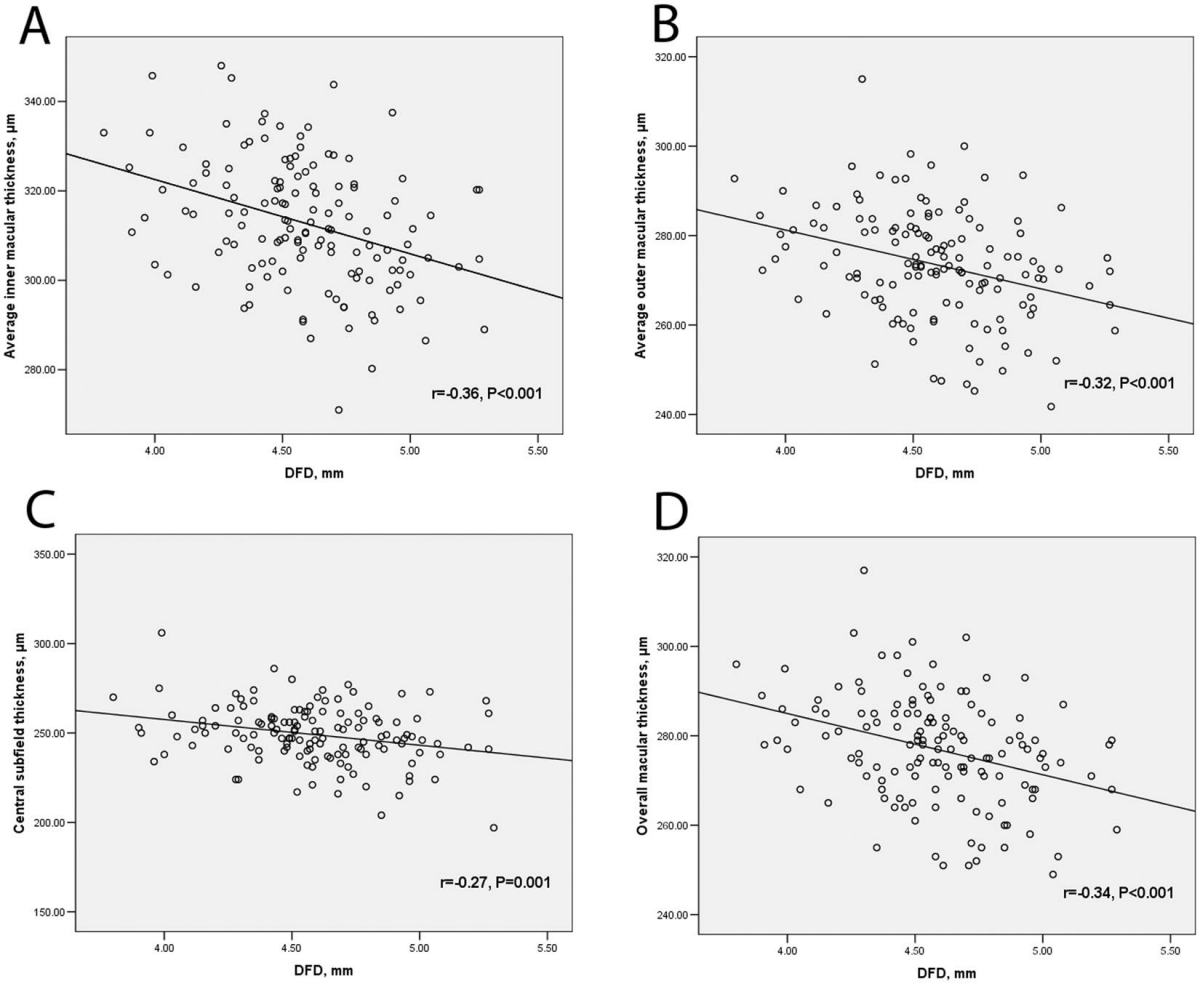
Scatter plots of DFD versus average inner macular thickness (**A**), average outer macular thickness (**B**), central subfield thickness (**C**), and overall macular thickness (**D**).

Table 2 demonstrates the associations between the macular thickness parameters and the ocular factors (including DFD, axial length, and refractive error). DFD was significantly correlated with all macular thickness parameters at a Bonferroni-corrected P value of 0.004 (0.05/12), with r ranging from −0.27 to −0.47, except for the temporal outer (r = −0.15, P = 0.089) and inferior outer(r = −0.22, P = 0.011) macular thickness. Axial length was mainly associated with the outer macular thickness parameters. This was less clear for the refractive error. Figure 2 shows the correlation between DFD and the summary macular thickness parameters. Age (r ranging from −0.08 to 0.06, all P > 0.3), signal strength (r ranging from −0.05 to 0.06, all P > 0.5), and visual field mean deviation (MD; r ranging from 0.01 to 0.11, all P > 0.1) did not correlate significantly with any of the macular parameters (within this group of subjects with a limited age range, signal strength range, and healthy eyes, that is, limited MD range). Eyes with β-PPA showed a thinner average macular thickness (273.7 versus 280.2 μm; P = 0.001) and a greater DFD (4.63 versus 4.53 mm; P = 0.002), compared to eyes without β-PPA. Table 3 presents the multivariable analysis. DFD, axial length, and gender were significantly associated with the average inner and outer macular thickness and the overall macular thickness; age and signal strength were not significant in the initial multivariable model and removed from the final model. Disc area and the presence of β-PPA were not significantly associated with any of the macular thickness measurements. Both DFD and gender were significantly associated with the central subfield thickness. There was no significant relationship between axial length and the central subfield thickness.

**Table 2:** Associations between ocular factors and macular thicknesses – univariable analysis (n = 138).

	Disc-fovea distance		Axial length		Refractive error	
	r	P	r	P	r	P
Superior inner macular thickness	−0.37	<0.001	−0.09	0.32	0.08	0.36
Nasal inner macular thickness	−0.35	<0.001	−0.02	0.81	0.09	0.29
Inferior inner macular thickness	−0.32	<0.001	−0.07	0.45	0.13	0.14
Temporal inner macular thickness	−0.31	<0.001	−0.03	0.74	0.11	0.20
**Average inner macular thickness**	−0.36	<0.001	−0.05	0.55	0.11	0.22
Superior outer macular thickness	−0.30	<0.001	−0.28	0.001	0.12	0.17
Nasal outer macular thickness	−0.47	<0.001	−0.09	0.32	0.001	1.0
Inferior outer macular thickness	−0.22	0.011	−0.25	0.003	0.16	0.058
Temporal outer macular thickness	−0.15	0.089	−0.28	0.001	0.22	0.012
**Average outer macular thickness**	−0.32	<0.001	−0.23	0.006	0.13	0.14
**Central subfield thickness**	−0.27	0.001	0.17	0.045	−0.05	0.60
**Overall macular thickness**	−0.34	<0.001	−0.19	0.024	0.11	0.19

**Table 3:** Associations between ocular factors and macular thicknesses – multivariable analysis, final models (n = 138).

	Average inner macular thickness (μm)		Average outer macular thickness (μm)		Central subfield thickness (μm)		Overall macular thickness (μm)	
	ß	P	ß	P	ß	P	ß	P
Disc-fovea distance (mm)	−18.4	<0.001	−15.9	<0.001	−14.6	0.001	−16.1	<0.001
Axial length (mm)	−2.31	0.023	−3.80	<0.001	—	NS	−3.30	<0.001
Gender (male)	8.83	<0.001	4.07	0.036	10.30	<0.001	4.27	0.025

In order to remove the confounding effect of ocular magnification on the OCT scan area and DFD, a subgroup analysis was performed in eyes with axial length between 24 and 25 mm. Table 4 shows the results. Again, DFD was strongly associated with macular thickness; a larger DFD implies a thinner macula.

**Table 4:** Associations between DFD and macular thicknesses (in μm) in subjects with axial length between 24 and 25 mm – multivariable analysis, final models (n = 31).

	ß	P
Average inner macular thickness	−32.1	<0.001
Average outer macular thickness	−25.7	<0.001
Central subfield thickness	−30.5	<0.001
Overall macular thickness	−27.2	<0.001

## Discussion

Disc-fovea distance has a significant inter—individual variability in healthy myopic eyes and is significantly associated with macular thickness. Eyes with a greater DFD have a lower average macular thickness and a lower central subfield thickness, independent of axial length and gender.

Variability in macular thickness in healthy eyes has been described previously^13–25^. Several factors, including age, axial length, and gender, have been reported to be associated with macular thickness^13–25^. A lower macular thickness has been observed in myopic eyes^13,16,18^, an effect that could be explained by stretching of the globe. Related to this, it has also been reported that a greater DFD is associated with a longer axial length^26^. Together this could suggest that the observed relationship between macular thickness and DFD is just an epiphenomenon of myopia. However, we found that DFD was independently associated with macular thickness, that is, not (only) through the effect of axial length. Macular thickness decreased typically 20–30 μm per mm increase in DFD (Tables 3 and 4; these analyses were adjusted for axial length), which corresponds to approximately 10 um per standard deviation of DFD (Table 1)^26^. As a result, a clinical assessment of macular thickness can be improved significantly by taking DFD into account. Importantly, it is easy to take DFD into account: unlike axial length, you don’t have to measure it separately because it can be measured from the OCT scan itself. Currently this can be done by using the built-in manual measurement tool of the OCT device; in the future this could be added to the software and normative database.

Apart from the stretching as described in the previous paragraph, there is another possible explanation is the difference in scan area for the macular measurements for eyes with different DFD. In eyes with greater DFD, the fovea is farther away from the optic disc, which implies that the OCT scan area (centered at the fovea) is farther away from the optic disc (for a given axial length). According to previous histological and imaging studies^28,29^, the RNFL is thinner farther from the optic disc than it is closer to the optic disc margin. Thus, one would expect to find that macular thickness is lower in eyes with greater DFD. On the other hand, the retinal ganglion cell layer becomes thicker towards the fovea. As macular thickness data for individual layers are not available for the 6 × 6 mm region of the Cirrus OCT, further studies are warranted.

Regional variations of the association between axial length and macular thickness have been described in previous studies^13,15–19^. We also found region-specific correlations between axial length and macular thickness (Table 2). However, this could be an artifact arising from the axial length related ocular magnification^30^. For the OCT device used in the current study, the default axial length is set to 24.46 mm^31^. Thus, due to ocular magnification, the scan area would be different from 6 × 6 mm in an eye with a shorter or longer axial length^30,32,33^. With the OCT device used in this study, it is impossible to obtain macular thickness data for an ocular magnification adjusted scan area. To reduce the confounding effect of axial length, we evaluated the correlation between DFD and macular thickness in a subgroup of subjects with a very narrow axial length range. A similar effect of DFD on macular thickness was observed in the multivariable analysis in this subgroup, indicating that DFD is indeed associated with macular thickness, independent of ocular magnification and other covariates.

It has been shown that myopic eyes have a steeper posterior retinal curvature than emmetropic eyes^34^. Thus, the DFD, based on two-dimensional images, may be underestimated in myopic eyes, especially in eyes with a posterior staphyloma^35^. Of note, most of the eyes included in present study were not high myopic eyes (the mean refractive error was −5.12 D). Moreover, eyes with myopic macular degeneration including a posterior staphyloma were excluded from the study. Importantly, the results of our subgroup analysis (Table 4) indicate that the uncovered influence of DFD on macular thickness cannot be a spurious effect of the steeper posterior retinal curvature artifact.

The association between gender and macular thickness has been reported in previous studies. Males have been found to have a greater sectoral and overall macular thickness compared to females^14,15,17,20,21^. Consistent with the previous reports, we found that males have a greater macular thickness after adjusting for DFD and other covariates. It has been reported that eyes with β*-*PPA tend to have a thinner macular thickness^36^. In line with previous studies^26,36^, we found that eyes with β*-*PPA had a thinner macular thickness and a greater DFD. In the multivariable analysis, however, the presence of β*-*PPA was not associated with macular thickness. Previous studies have reported that age is significantly correlated with macular thickness^14,15,17,20–22^. In the present study, we did not detect an association between age and any of the macular thickness parameters. One possible explanation is that the age range in our study is relatively narrow. Most of the subjects included in our study were young myopic subjects (Table 1). It has been reported that the image quality of OCT scans affects the observed retinal layer thicknesses^37–39^, and image quality decreases with an increase in myopia^40^. Consistent with Lee *et al*.'s study^40^, we found a negative correlation between signal strength and axial length (r = −0.32, P < 0.001). However, we did not detect a significant relationship between image quality and macular thickness. This is presumably due to the fact that only OCT scans with a high image quality (minimum signal strength was set at 7; 90% of the scans had a signal strength of 8 or better) were included in the current study, which limits the variability of this variable.

There are limitations in the present study. One limitation is that only young myopic subjects of the same ethnicity (all were Chinese) were included. Thus, the current findings may not apply to other populations. Racial differences in macular thickness have been reported^41^. Further studies are needed to evaluate the association between DFD and macular thickness in other populations and for a wider age range.

In conclusion, a significant inter-subject variability exists in DFD and macular thickness in healthy myopic eyes. Eyes with a greater DFD have a lower macular thickness, independent of axial length. A clinical assessment of macular thickness should always be interpreted in the context of DFD.

## Methods

### Subjects

One hundred and forty seven Chinese healthy myopic subjects with a spherical equivalent less than −0.5 diopters (D) were consecutively recruited from the refractive surgery clinic of Joint Shantou International Eye Center. All the included subjects underwent a full ophthalmic examination including the measurement of visual acuity and intraocular pressure (IOP), refraction, perimetry (see below), and a dilated stereoscopic fundus examination including assessment of the presence of β-zone peripapillary atrophy (β-PPA). Axial length was determined using the IOL Master (Carl-Zeiss Meditec, Dublin, CA). Only measurements with a signal-to-noise ratio above 2.0 were included. We recorded five measurements and used the mean value for analysis. None of the included eyes had any concurrent ocular disease other than a refractive error. One eye from each subject was included for analysis; if both eyes were eligible, a random eye (based on a computer-generated randomization list) was selected. Subjects were excluded if the best corrected visual acuity was less than 20/40, the IOP over 21 mmHg, if they had a family history of glaucoma, or if they had a history of intraocular surgery, myopic macular degeneration, glaucoma, refractive surgery, neurological disease, or diabetes. The study was approved by the ethical committee of Joint Shantou International Eye Center with written informed consent obtained from each subject before enrolment. The present study followed the tenets of the declaration of Helsinki.

### Visual field testing

Visual field testing was performed with standard automated perimetry using the 24–2 grid and the SITA standard strategy (Humphrey Field Analyzer II; Carl Zeiss Meditec, Inc.). Only reliable visual field tests (with fixation loss less than 20% and false positive and negative less than 10%) were included in the study. All visual field tests of the included subjects had a pattern standard deviation (PSD) P > 5% and were within normal limits in the glaucoma hemifield test (GHT).

### Optical coherence tomography

All eyes received macular and optic disc imaging using the Cirrus High Definition OCT (software version 5.0.0.326; Carl Zeiss Meditec, Dublin, CA). The scan speed for this spectral-domain OCT is 27,000 A-scans per second and the axial resolution is 5 μm^42^. Both the Optic Disc Cube 200 × 200 protocol and the Macular Cube 200 × 200 protocol were performed. Disc area generated by the Optic Disc Cube 200 × 200 protocol was recorded for subsequent analysis. The Macular Cube 200 × 200 protocol was used for macular thickness measurements in an area of 6 × 6 mm. Eye movements were checked by reviewing the line-scanning ophthalmoscope (LSO) fundus images. Images with misaligned vessels within the scanning area were excluded and retaken. All the included images had minimum signal strength of 7. Images with segmentation errors were excluded from the analysis (3.5%).

The Cirrus HD-OCT software was used to generate retinal thickness maps. The mean thickness was determined for nine retinal subfields in three concentric circles (with diameters of 1, 3, and 6 mm) centered at the fovea, as defined by the Early Treatment Diabetic Retinopathy Study (ETDRS). The retinal subfields in the retinal thickness map are central, inner superior, inner nasal, inner inferior, inner temporal, outer superior, outer nasal, outer inferior, and outer temporal. The central subfield thickness was defined by the innermost 1 mm diameter circle while the inner and outer subfields were bounded by the 1 and 3 and 3 and 6 mm diameter circles, respectively. Apart from the nine retinal subfields, the average of the four-quadrant macular thicknesses in the inner and outer rings was also calculated as average inner macular thickness and average outer macular thickness, respectively. Overall macular thickness in the entire grid area was also recorded from the retinal thickness maps.

### Measurement of DFD

**Figure 3:**
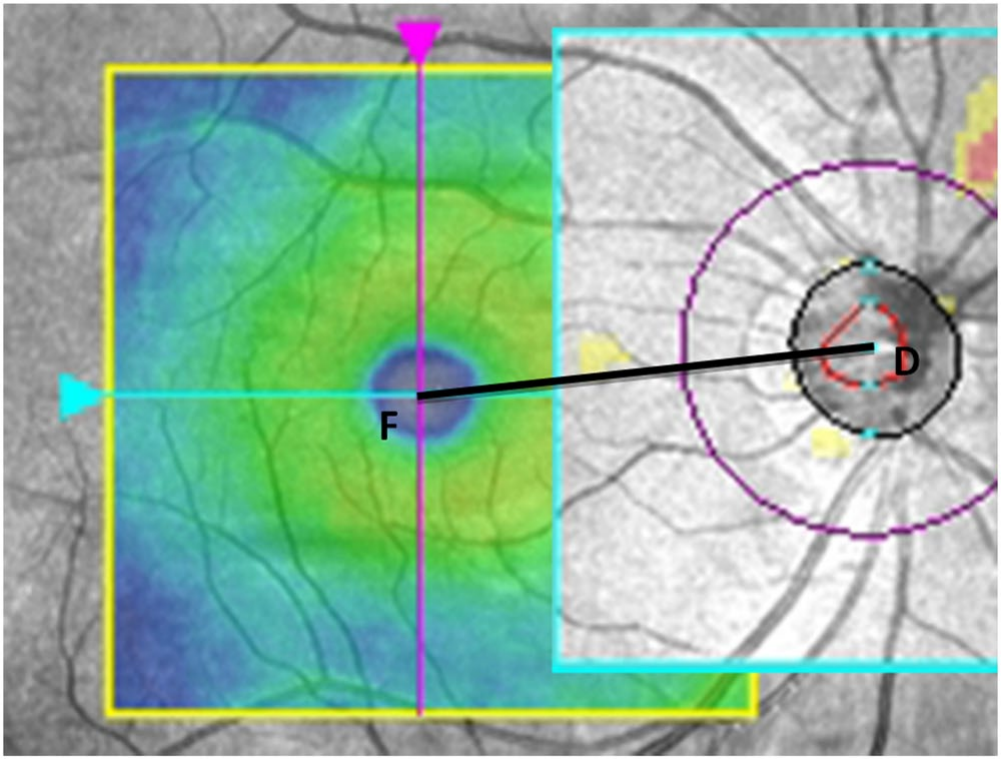
Measurement of optic disc-fovea distance (DFD) on SLO fundus image. The fovea (point F) was automatically detected by the OCT software on the SLO fundus image with the macular color thickness map. The enface optic disc image (RNFL deviation map) with the optic disc center labelled (point D) by the OCT software was manually registered to the SLO fundus image with Illustrator CS4 software using the retinal vessels as reference. Measurement of DFD (distance from F to D) was performed with ImageJ software based on the coordinates of the fovea and the center of the optic disc.

Measurement of DFD was performed with ImageJ software (available in the public domain at http://rsbweb.nih.gov/ij/; www.nih.gov, National Institutes of Health, Bethesda, MD, USA), based on the coordinates of the fovea and the center of the optic disc. For this purpose, we used carefully registered OCT projection images, as illustrated in Fig. 3. We used the LSO fundus image with the macular color thickness map overlay to localize the fovea. The fovea was automatically detected by the OCT software. Subsequently, the enface optic disc image (RNFL thickness deviation map) from the RNFL thickness report was exported and manually registered to the LSO fundus image with Illustrator CS4 software (Adobe Systems Inc., San Jose, California). For this, the transparency of the optic disc image was set to 50% to allow visualization of the underlying LSO fundus image; the retinal vessel trajectories were used as a reference. The optic disc center and the disc margin were determined by the built-in software and shown on the RNFL thickness deviation map, based on the margin of Bruch’s membrane. The DFD was then measured by using the ImageJ software on the overlaid images^43,44^.

### Ocular magnification and subgroup analysis

The actual size of objects at the level of the retina may differ from the reported size, depending on the axial length related ocular magnification. We could adjust the DFD for this magnification, but the built-in software does not adjust the macular area over which the thicknesses and volume are reported. For that reason, we did not adjust DFD for ocular magnification in our main analysis. We also studied the effect of DFD on macular thickness in a subgroup of subjects with a very narrow axial length range, in order to circumvent the confounding effect of ocular magnification. For this analysis, we restricted the axial to a 1 mm range, 24 to 25 mm, centered around the default axial length of OCT of 24.46 mm^31^.

### Statistical analysis

Univariable (Pearson’s or Spearman’s correlation coefficient depending on the distribution) and multivariable analysis (multiple linear regression) were performed to determine the effects of axial length/refractive error, age, gender, image quality, and DFD on the macular thickness. A P value of 0.05 was considered statistically significant; Bonferroni correction was applied if applicable. In the multivariable analysis, we used backward stepwise regression by including initially all variables and subsequently removing, one at a time, those variables with P > 0.05, starting with the variable with the highest P value. The statistical analyses were performed by using the SPSS software (ver. 17.0; SPSS Inc, Chicago, IL).
